# Typical Kawasaki Disease Presenting With Pancreatitis and Bilateral Parotid Gland Involvement: A Case Report and Literature Review

**DOI:** 10.3389/fped.2018.00090

**Published:** 2018-04-11

**Authors:** Matteo Botti, Giorgio Costagliola, Rita Consolini

**Affiliations:** Section of Pediatrics Immunology and Rheumatology, Department of Pediatrics, University of Pisa, Pisa, Italy

**Keywords:** pancreatitis, Kawasaki disease, fever of unknown origin, parotitis, hyperamylasemia

## Abstract

We describe the case of a 3-year child in which pancreatic and parotid gland involvement preceded the development of the classical clinical phenotype of a typical Kawasaki disease (KD). The child was referred to the Emergency Department with a story of 3 days of continuous fever associated with abdominal pain and bilaterally swelling in the parotid regions; laboratory evaluation identified markedly increased levels of total amylase, pancreatic amylase, lipase, and transaminase, and diagnosis of pancreatitis was posed. After 9 days of fever and persistence of the clinical features, the classical signs of KD appeared, and the child was treated with intravenous immunoglobulins (IVIG), showing a dramatic response with complete resolution of the clinical picture. In this work, we reviewed the literature about gastrointestinal (GI) symptoms in KD, focusing on pancreatic and hepatic involvement. This analysis highlighted that, in case of fever associated with pancreatic inflammation, KD must be considered in the spectrum of differential diagnosis, and that GI involvement in KD is frequently associated with an incomplete response to IVIG treatment.

## Background

Kawasaki disease (KD) is a multisystemic inflammatory disease of childhood, histologically characterized by a vasculitis of medium-sized vessels. The vascular inflammation usually affects coronary arteries, but the disease can potentially involve other vascular districts, including abdominal vessels. Current literature reports many studies describing liver abnormalities in KD, but there are only a few works investigating the association between KD and pancreatitis or parotitis. In this article, we report the case of a typical KD presenting with severe pancreatitis, bilateral parotitis, and hepatic dysfunction, and we review the literature about gastrointestinal (GI) involvement in KD.

## Case Presentation

A 3-year-old Italian child was referred to our hospital after 3 days of continuous fever associated with bilateral swelling of the parotid regions. The symptoms started about 15 days before with dry cough, initially treated with salbutamol aerosol and oral betamethasone for suspected viral laryngitis, and subsequently with oral antibiotics (cefixime) for the development of pharyngitis and fever. He was first addressed to the Pediatric Emergency Department with a bilateral swelling of the parotid region as well as a bilateral cervical lymphadenopathy. The body temperature was 39.5°C. Abdomen showed absence of tenderness or muscular defense, and Blumberg sign was negative. Examination of the skin revealed an erythematous macular rash mainly spreading in the trunk, and the remaining physical assessment was negative, except for the finding of a mild systolic murmur (II/VI Levine Scale). Laboratory investigations showed white blood count (12.3 × 10^3^/mL, 70% neutrophils), elevated levels of total amylase (502 U/L), transaminases, aspartato-transaminase (AST: 441 IU/L), alanine aminotransferase (ALT: 212 IU/L), glutamic-oxalacetic transaminase (60 U/L), C-reactive protein (CRP: 7.9 mg/L), and procalcitonin (0.25 ng/mL), while electrolytes, renal function, and other parameters resulted normal. Table 1 summarizes laboratory findings at presentation and during hospitalization.

**Table 1:** Laboratory findings at clinical presentation and during hospitalization.

Parameter (normal values)	Day 1	Day 3	Day 4	Day 9	Day 12	Day 19
Hemoglobin (10.5–15.5 g/dL)	10.6	10.7	10.2	10.2	10.2	9.1
Platelet count (150–450 × 10^3^/mL)	421	362	448	524	692	609
Leukocyte count (5.0–14.0 × 10^3^/mL)	12.32	11.97	11.61	6.45	9.95	10.16
C-reactive protein (<5 mg/L)	7.9	33	29	21.6	11.7	7.2
Amylase (28–100 IU/L)	502	1,369	1,297	261	178	116
Lipase (<60 IU/L)	43	905	1,796	107	155	111
Pancreatic amylase (15–53 IU/L)	35	340	918	80	115	100
Aspartato-transaminase (<40 IU/L)	441	272	55	25	30	33
Alanine aminotransferase (<41 IU/L)	212	240	112	25	18	17
Total bilirubin (<1.2 mg/dL)	0.14	0.24	0.21	0.23	0.27	0.24
Conjugated bilirubin (<0.3 mg/dL)	0.09	0.15	0.14	0.13	0.19	0.13
Protein count (5.6–7.5 g/dL)	6.2	6.7	6.4	6.7	6.6	8
Creatinine (0.24–0.41 mg/dL)	0.56	0.32	0.38	0.45	0.47	0.41
Urine analysis (normal)	Normal	Normal	Normal	Normal	Normal	Normal

The day after the admission to our Pediatric Department, the laboratory analysis showed a dramatic increase of total amylase (1.369 IU/L), pancreatic amylase (340 IU/L), and lipase (905 IU/L), and the abdomen developed light tenderness. Consequently to the association of abdominal pain and elevated levels of pancreatic enzymes, the diagnosis of acute pancreatitis, defined as “not severe” according to DeBanto pediatric score (1) (2 points), was posed. The patient started a strict diet without lipids (1,100 kcal/day, about 70–75 kcal/kg/day), and hydration with intravenous saline (0.9%) and dextrose solution (5%).

At the third day of hospitalization, the abdomen and neck ultrasound showed a bilateral enlargement of lymph nodes (maximum diameter: 2 cm) in the lateral cervical region, and mildly enlarged lymph nodes with preserved architecture and morphology next to the parotid gland; the examination of the liver, the gallbladder, and the pancreas showed absence of pathological findings. The laboratory investigation for viral acute infection (adenovirus, parvovirus, cytomegalovirus, mumps, Epstein–Barr, and Coxsackie virus, performed by ELISA and Polymerase Chain Reaction) were negative; the patient, not previously vaccinated against Mumps, showed positivity for anti-Mumps IgG titer, in absence of anti-Mumps IgM, suggestive for previous Mumps infection.

**Figure 1:**
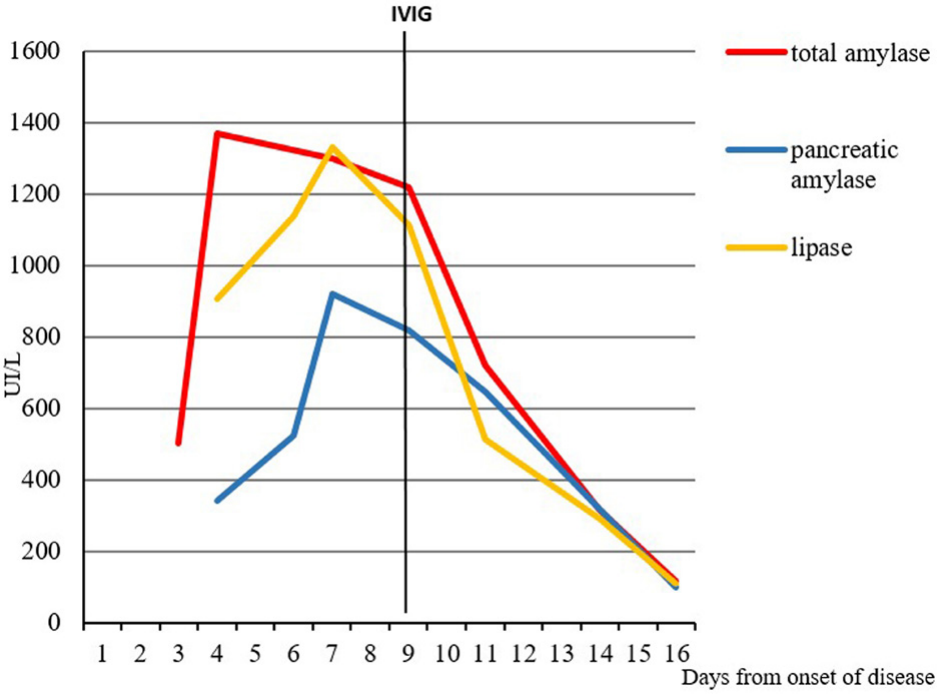
Relationship between the administration of intravenous immunoglobulins (IVIG) and levels of pancreatic enzymes.

Symptoms and laboratory findings persisted without significant improvement of child’s clinical condition. At the fourth day after recovery, the total values of amylase, pancreatic amylase, lipase, and CRP were still elevated (respectively, 1.297 IU/L, 918 IU/L, 1.330 IU/L, and 30.3 mg/L), whereas the transaminases were in reduction (AST 29 IU/L, ALT 60 IU/L). Despite the administration of paracetamol, the fever persisted reaching peaks of 40°C, and the general conditions worsened: the child appeared extremely suffering, irritable, and complained of diffuse abdominal pain, without clinical signs of acute peritonitis. At the sixth day of hospitalization (continuous fever lasting for 9 days), appeared some mucocutaneous manifestations, strongly suggestive for the diagnosis of KD: peri-orbital and hand mild edema, new erythematous macular rash, conjunctival injection without exudate and markedly fissured erythematous lips. In addition, the systolic heart murmur was more appreciable (III/VI Levine scale) than at the presentation. The diagnosis of KD with classic clinical criteria was confirmed (2). Echocardiography demonstrated absence of both functional heart involvement and coronary aneurysms. The therapy was started with intravenous immunoglobulins (IVIG), at a dose of 2 g/kg in 12 h, and acetylsalicylic acid (80 mg/kg/day in four doses), with strongly evident effects in the first 3–4 h: the body temperature dramatically decreased under 37.5°C and the general conditions rapidly improved. The child was kept on a restricted fat diet and treatment with acetylsalicylic acid was continued showing a progressive resolution of the clinical picture, including abdominal pain, mucocutaneous signs, lymphadenopathy, and parotid swelling. The laboratory tests at discharge (after 19 days of hospitalization) evidenced a reduction of total amylase (116 IU/L), pancreatic amylase (100 IU/L), lipase (111 IU), and CRP (7.2 mg/L). The trend of total amylase, pancreatic amylase, and lipase, and its relationship with the administration of IVIG, is shown in Figure 1.

The child no longer complained of abdominal pain and no longer appeared irritable, except for the hunger due to the prolongation of the diet. Echocardiographic follow-up was performed until the discontinuation of antithrombotic therapy with acetylsalicylic acid (at dose of 4 mg/kg for 6 weeks after discharge), always showing absence of pathological findings.

## Discussion

Kawasaki disease is a generalized multisystemic inflammatory disease of unknown etiology characterized by a vasculitis involving medium-sized arteries (2).

We report a case of typical KD arising with fever and contextual inflammation of pancreas, parotids, and liver revealed by an increase of total and pancreatic amylase and transaminases.

In the described case, the simultaneous presence of pancreatic and parotid gland involvement initially led to the clinical suspect of an infectious process, particularly mumps, but the elevated title of anti-mumps IgG antibodies, the absence of anti-mumps IgM antibodies, and the negativity for other serology’s opened the spectrum of further differential diagnoses.

The classic criteria of KD appeared only 6 days after the hospitalization (corresponding to 9 days of continuous fever), and the most evident signs of KD in this case were the strongly fissured lips, a mild conjunctivitis, the skin rash, and the cervical lymphadenopathy.

Despite the limitation deriving by the description of a single case, this work adds some important issues, useful for correctly approaching GI involvement in KD.

In fact, although GI symptoms are not included in the classic diagnostic criteria of KD, they are often associated with the disease. In particular, hepatic dysfunction and hydrops of the gallbladder are common in children with KD and several studies reported liver function abnormalities (3–5). About 40% of patients with KD have been reported to have subclinical liver involvement, defined by the elevation of one or both liver transaminases and/or GGT (6), which are in line with the findings of our case. Although hepatic disease is not a significant cause of morbidity or mortality in KD, a recent meta-analysis by Liu et al. reported that liver abnormality was significantly associated with IVIG unresponsiveness in KD patients (7).

On the contrary, there are few studies in literature reporting pancreatitis and parotitis as phenomenon related with KD, even though the real incidence of these conditions in KD is unknown. The association between parotid gland inflammation and KD was previously described in a unique case in a 3-month-old female infant; in this report, typical KD features appeared after 5 days of hospitalization, and the disease was associated with multiple coronary aneurysms and unresponsiveness to IVIG (8).

Current literature reports only nine cases of KD-associated pancreatitis. The first two cases were reported by Stoler et al. in 1987, both evidencing pancreatic involvement in the second week of disease (9), and both not treated with the IVIG infusion; one of these patients developed multiple coronary aneurysms.

Lanting et al., similar to our report, described a case of a 5-year-old patient, in which pancreatic involvement preceded the other manifestations of KD and reached complete responsiveness to IVIG therapy (10).

By analyzing a Japanese cohort of KD patients, Asano et al. found that 12 of 138 cases presented with elevated serum amylase levels (>170 IU/L), mainly deriving from the salivary glands rather than the pancreas (11). In only a case out of these 12 ones, the pancreatic inflammation was confirmed by an increase of pancreatic amylase isozyme.

A case of a 6-year-old boy showing an increase of serum amylase (168 IU/L) and transaminases associated with hydrops of the gallbladder and edematous pancreas as revealed by ultrasound was reported as onset picture of atypical KD (12).

The more recent literature reports two cases of KD who developed, after several days of illness, common bile duct stenosis and symptomatic pancreatitis, following the administration of two consecutive doses of IVIG (13).

Despite the complete response to IVIG achieved by our patient, the analysis of literature highlighted that the unresponsiveness to a first dose of IGIV is common in patients with KD-associated pancreatitis. Interestingly, there is a report on the case of an IVIG-resistant KD in whom the administration of Infliximab resulted in clinical improvement and resolution of acute pancreatitis (14). On the basis of current information (2), the use of Infliximab, a monoclonal antibody that binds the cytokine TNF-α, in KD therapy is a safe procedure that reduce significantly the systemic levels of inflammation and could be considered a “rescue therapy” in IGIV resistance KD patient, that have already received the second dose of IGIV or the prednisone. Therefore, the administration of Infliximab may be considered reasonable, like second-line or third-line therapy, also in the cases with severe inflammatory involvement of pancreas or other organs.

In the Table 2, we summarize the main parameters derived from the detailed synthesis of published cases with pancreatic involvement in KD. We underline the predominance of male gender, whereas the age of presentation is mutable, with predilection of later age, with a mean age of 6 years at diagnosis.

**Table 2:** Summary of published data on Kawasaki disease with pancreatitic involvement (case reports).

Reference	Age (years)	Gender	Pancreatitis at the clinical presentation	Peak level of serum total amylase (IU/L)	Time of peak serum amylase from onset of disease (days)	Peak level of serum lipase (IU/L)	Cardiac involvement	Timing of administration IVIG after onset of fever (days)
Stoler et al. (9)	5	M	No	197	9th	U	Yes	ND
Stoler et al. (9)	16	M	No	356	25th	U	No	ND
Lanting et al. (10)	5	M	Yes	1,143	3rd	633	No	7th
Asano et al. (11)	1	M	Yes	407	14th	89	No	6th
Prokic et al. (12)	6	M	Yes	168	5th	95	No	7th
Cherry et al. (13)	6	M	No	193	30th	420	Yes	5th^a^
Cherry et al. (13)	3	M	No	465	15th	3,224	Yes	4th^a^
Jimenez-Fernandez and Tremoulet (14)	10	F	Yes	U	U	U	No	9th^b^
Present case (2018)	3	M	Yes	1,369	5th	1.330	No	9th

*^a^Needed of second doses of IVIG*.

*^b^Needed of infliximab*.

*U, unknown; ND, not done; IVIG, intravenous immunoglobulins*.

The analysis of literature points out that pancreatitis can precede the onset of the disease, be part of the classical or atypical clinical picture of KD, or paradoxically appears several days after the administration of IVIG. Abdominal pain is the most common symptom of pancreatic involvement, although some patients are asymptomatic exhibiting only a lipasemia.

Moreover, it is important to emphasize that in patients who have been reported to develop pancreatitis after clinical onset of KD often suffer from cardiac affections suggesting that patients with late pancreatic involvement deserve a strict clinical and echocardiographic follow-up. However, in most of the cases described in literature, patients had early pancreatic involvement, that was not associated with the development of heart disease.

The pathogenesis of hepatic and pancreatic alterations in KD has not yet been completely elucidated. In a study, the results of the autopsies performed on KD patients, who had died before the historical introduction of IVIG in KD therapy, revealed the presence of pancreatitis in 22% of the cases, and 53% of subjects overall had inflammation of the pancreatic duct (15). It has been supposed that the widespread inflammation typical of this disease could involve unusual organs such as the pancreas, although pancreatic inflammation often remains subclinical. Subsequently, microscopic examination confirmed the presence of vasculitis of the pancreas affecting medium-sized arteries, characterized by intimal inflammation, media destruction, and perivascular flogosis (16). Interestingly, a recent study reported that the infiltration damaging the pancreatic islets was characterized by presence of CD8+ T-lymphocytes and macrophages, similar to that one observable in the aneurysms of the coronary arteries (17). These data support the conclusion that pancreatic inflammation in KD is caused by immune-mediated vascular involvement, differently to the other forms of pancreatitis, including the viral ones, in which the organ damage derives mainly from the direct destruction of acinar cells or from the intrapancreatic activation of enzymes (18).

## Concluding Remarks

We focused on the involvement of liver, parotid gland, and particularly of pancreas in patients affected by KD. The analysis of our case report points out that in case of persistence over 5 days of unexplained fever associated with clinical or laboratory signs of pancreatic and hepatic involvement, KD should be included in the spectrum of differential diagnosis, also in the absence of the classical diagnostic criteria. Our review of literature highlighted the association of hepatopancreatic involvement with the unresponsiveness to IVIG, and evidenced that patients with late presentation of pancreatitis often develop cardiac alterations.

## Ethics Statement

Written informed consent for the publication of this case report and figures was obtained from the parents.

## Author Contributions

MB and GC performed the literature review and wrote the manuscript. RC critically reviewed the manuscript.

## Conflict of Interest Statement

The authors declare that the research was conducted in the absence of any commercial or financial relationships that could be construed as a potential conflict of interest.
